# Supplementary material for: Computational Profiling of Monoterpenoid Phytochemicals: Insights for Medicinal Chemistry and Drug Design Strategies
Source: Int J Mol Sci. 2025 Aug 8;26(16):7671. doi: 10.3390/ijms26167671 (PMC12386793; doi:10.3390/ijms26167671)
Supplement: Supplementary file 1 [file ijms-26-07671-s001.zip › Table S2.pdf]

Table S2

Prediction of targets that molecule CID 139587999 has 70-100% cutoff probability of interaction using TargetNet Server

Targets	Prob (%)
Polyphenol oxidase 2	100%
Acetylcholinesterase	100%
Androgen receptor	100%
Carbonic anhydrase 5B, mitochondrial	100%
Carbonic anhydrase 4	100%
Carbonic anhydrase 6	100%
Carbonic anhydrase 5A, mitochondrial	100%
Arachidonate 15-lipoxygenase	100%
Nitric oxide synthase, inducible	100%
Glucocorticoid receptor	100%
Carbonic anhydrase 7	100%
Estrogen receptor	100%
Receptor-interacting serine/threonine-protein kinase 2	99%
Muscarinic acetylcholine receptor M2	99%
Sphingosine 1-phosphate receptor 2	99%
Muscarinic acetylcholine receptor M4	98%
Muscarinic acetylcholine receptor M1	97%
Corticosteroid 11-beta-dehydrogenase isozyme 2	96%
Steroid 17-alpha-hydroxylase/17,20 lyase	96%
Perilipin-1	96%
M-phase inducer phosphatase 2	93%
COUP transcription factor 2	90%
DNA dC->dU-editing enzyme APOBEC-3A	88%
Cannabinoid receptor 2	87%
Carbonic anhydrase 12	86%
Cytochrome P450 2C19	83%
Estrogen receptor beta	83%
Carbonic anhydrase 9	82%
G-protein coupled bile acid receptor 1	78%
Neuronal acetylcholine receptor subunit alpha-4	77%
Alkaline phosphatase, tissue-nonspecific isozyme	76%
Prostaglandin G/H synthase 1	76%
Carbonic anhydrase 13	71%

Prediction of targets that molecule CID 98642738 has 70-100% cutoff probability of interaction using TargetNet Server

Targets	Prob (%)
Corticosteroid 11-beta-dehydrogenase isozyme 2	100%
Steroid 17-alpha-hydroxylase/17,20 lyase	100%
Nitric oxide synthase, inducible	100%
Polyphenol oxidase 2	100%
Acetylcholinesterase	100%
Androgen receptor	100%
Corticosteroid 11-beta-dehydrogenase isozyme 1	100%
Muscarinic acetylcholine receptor M1	100%
Glucocorticoid receptor	100%
Carbonic anhydrase 5B, mitochondrial	100%
M-phase inducer phosphatase 2	99%
5-hydroxytryptamine receptor 1E	99%
Dipeptidyl peptidase 4	99%
Carbonic anhydrase 5A, mitochondrial	99%
Muscarinic acetylcholine receptor M4	98%
Cannabinoid receptor 2	98%
Arachidonate 15-lipoxygenase	97%
Aromatase	97%
Heat shock protein HSP 90-alpha	97%
Muscarinic acetylcholine receptor M2	96%
Carbonic anhydrase 7	96%
Carbonic anhydrase 6	96%
Sphingosine 1-phosphate receptor 2	96%
Carbonic anhydrase 4	95%
Dipeptidyl peptidase 8	95%
Carbonic anhydrase 14	94%
COUP transcription factor 2	93%
Alkaline phosphatase, tissue-nonspecific isozyme	90%
Cannabinoid receptor 1	89%
Alpha-2C adrenergic receptor	89%
Cytochrome P450 2C19	88%
Neuronal acetylcholine receptor subunit alpha-4	85%
DNA dC->dU-editing enzyme APOBEC-3A	79%
Carbonic anhydrase 12	76%
Dipeptidyl peptidase 2	74%
3-oxo-5-alpha-steroid 4-dehydrogenase 2	74%
Toll-like receptor 9	73%

Prediction of targets that molecule CID 156582074 has 70-100% cutoff probability of interaction using TargetNet Server

Targets	Prob (%)
Steroid 17-alpha-hydroxylase/17,20 lyase	100%
Androgen receptor	100%
Acetylcholinesterase	100%
Alpha-2C adrenergic receptor	100%
Nitric oxide synthase, inducible	100%
Cathepsin G	100%
Muscarinic acetylcholine receptor M4	100%
Muscarinic acetylcholine receptor M1	99%
5-hydroxytryptamine receptor 1E	98%
Cannabinoid receptor 2	94%
Alpha-2B adrenergic receptor	90%
Potassium voltage-gated channel subfamily A member 3	90%
Arachidonate 15-lipoxygenase	89%
Corticosteroid 11-beta-dehydrogenase isozyme 2	88%
Cocaine esterase	85%
DNA dC->dU-editing enzyme APOBEC-3A	82%
Sphingosine 1-phosphate receptor 2	79%

Prediction of targets that molecule CID 139205363 has 70-100% cutoff probability of interaction using TargetNet Server

Targets	Prob (%)
Corticosteroid 11-beta-dehydrogenase isozyme 2	100%
Steroid 17-alpha-hydroxylase/17,20 lyase	100%
Androgen receptor	100%
Corticosteroid 11-beta-dehydrogenase isozyme 1	100%
Alpha-2C adrenergic receptor	100%
Cocaine esterase	100%
Alpha-2B adrenergic receptor	100%
Nitric oxide synthase, inducible	100%
Acetylcholinesterase	100%
DNA dC->dU-editing enzyme APOBEC-3A	100%
5-hydroxytryptamine receptor 1E	100%
Muscarinic acetylcholine receptor M4	100%
Squalene synthase	98%
Muscarinic acetylcholine receptor M3	94%
Cytochrome P450 2D6	93%
Sphingosine 1-phosphate receptor 2	93%
Kappa-type opioid receptor	92%
3-oxo-5-alpha-steroid 4-dehydrogenase 2	86%
Muscarinic acetylcholine receptor M1	80%

Prediction of targets that molecule CID 5362595 has 70-100% cutoff probability of interaction using TargetNet Server

Targets	Prob (%)
Nitric oxide synthase, inducible	100%
DNA dC->dU-editing enzyme APOBEC-3A	100%
Muscarinic acetylcholine receptor M4	100%
Plasminogen activator inhibitor 1	100%
Muscarinic acetylcholine receptor M2	100%
Sphingosine 1-phosphate receptor 5	100%
Aminopeptidase N	100%
Muscarinic acetylcholine receptor M3	99%
Nitric oxide synthase, brain	99%
Sphingosine 1-phosphate receptor 2	99%
Cannabinoid receptor 2	99%
Muscarinic acetylcholine receptor M1	99%
M-phase inducer phosphatase 2	99%
Polyphenol oxidase 2	98%
CHRNA7-FAM7A fusion protein	97%
COUP transcription factor 2	95%
Neuronal acetylcholine receptor subunit alpha-7	94%
Prostaglandin F2-alpha receptor	93%
Monoglyceride lipase	92%
Phenylethanolamine N-methyltransferase	92%
5-hydroxytryptamine receptor 1E	90%
5-hydroxytryptamine receptor 3A	86%
Heat shock protein HSP 90-alpha	77%

Prediction of targets that molecule CID 146683370 has 70-100% cutoff probability of interaction using TargetNet Server

Targets	Prob (%)
Nitric oxide synthase, inducible	100%
Acetylcholinesterase	100%
DNA dC->dU-editing enzyme APOBEC-3A	100%
M-phase inducer phosphatase 2	100%
Muscarinic acetylcholine receptor M2	98%
Neuronal acetylcholine receptor subunit alpha-4	87%
Prostaglandin F2-alpha receptor	85%
Liver carboxylesterase 1	83%
Muscarinic acetylcholine receptor M3	77%
Cocaine esterase	76%

Prediction of targets that molecule CID 101416067 has 70-100% cutoff probability of interaction using TargetNet Server

Targets	Prob (%)
3-oxo-5-alpha-steroid 4-dehydrogenase 2	100%
Steryl-sulfatase	100%
Androgen receptor	100%
Tyrosine-protein phosphatase non-receptor type 2	100%
Muscarinic acetylcholine receptor M2	100%
Cannabinoid receptor 2	100%
Carbonic anhydrase 12	100%
Steroid 17-alpha-hydroxylase/17,20 lyase	100%
Carbonic anhydrase 14	100%
Nitric oxide synthase, inducible	100%
Carbonic anhydrase 9	100%
Polyphenol oxidase 2	100%
Acetylcholinesterase	100%
Muscarinic acetylcholine receptor M4	100%
3-oxo-5-alpha-steroid 4-dehydrogenase 1	100%
Neuronal acetylcholine receptor subunit alpha-4	100%
Cocaine esterase	100%
Carbonic anhydrase 5B, mitochondrial	100%
Corticosteroid 11-beta-dehydrogenase isozyme 2	100%
Potassium voltage-gated channel subfamily A member 3	100%
Carbonic anhydrase 6	100%
M-phase inducer phosphatase 2	100%
Retinoic acid receptor alpha	100%
Carbonic anhydrase 5A, mitochondrial	100%
Liver carboxylesterase 1	100%
G-protein coupled bile acid receptor 1	99%
Aromatase	99%
Glucocorticoid receptor	99%
Arachidonate 15-lipoxygenase	99%
Carbonic anhydrase 4	99%
G-protein coupled receptor 35	99%
Muscarinic acetylcholine receptor M5	98%
Retinoic acid receptor gamma	98%
Tyrosine-protein phosphatase non-receptor type 1	97%
Muscarinic acetylcholine receptor M3	96%
DNA dC->dU-editing enzyme APOBEC-3A	96%
Retinoic acid receptor beta	94%
Corticosteroid 11-beta-dehydrogenase isozyme 1	94%
5-hydroxytryptamine receptor 1E	90%
Tubulin beta-2B chain	90%
Muscarinic acetylcholine receptor M1	88%
DNA (cytosine-5)-methyltransferase 1	88%
Sphingosine 1-phosphate receptor 2	85%
NAD-dependent protein deacetylase sirtuin-2	82%
Cytochrome P450 2C19	82%
