# Supplementary material for: Computational Profiling of Monoterpenoid Phytochemicals: Insights for Medicinal Chemistry and Drug Design Strategies
Source: Int J Mol Sci. 2025 Aug 8;26(16):7671. doi: 10.3390/ijms26167671 (PMC12386793; doi:10.3390/ijms26167671)
Supplement: Supplementary file 1 [file ijms-26-07671-s001.zip › Table S3.pdf]

Table S3 - Broader cutoff was applied to identify targets shared by 1, 2, 3, 4, 5, 6, or all 7 compounds.

Target Name	I	II	III	IV	V	VI	VII	Total
5-hydroxytryptamine receptor 1E	X		X	X	X		X	5
5-hydroxytryptamine receptor 3A					X			1
Aminopeptidase N					X			1
Cannabinoid receptor 2	X	X	X		X		X	5
CHRNA7-FAM7A fusion protein					X			1
COUP transcription factor 2	X	X			X			3
DNA dC->dU-editing enzyme APOBEC-3A	X	X	X	X	X	X	X	7
Heat shock protein HSP 90-alpha	X				X			2
Monoglyceride lipase					X			1
M-phase inducer phosphatase 2	X	X			X	X	X	5
Muscarinic acetylcholine receptor M1	X	X	X	X	X		X	6
Muscarinic acetylcholine receptor M2	X	X			X	X	X	5
Muscarinic acetylcholine receptor M3				X	X	X	X	4
Muscarinic acetylcholine receptor M4	X	X	X	X	X		X	6
Neuronal acetylcholine receptor subunit alpha-7					X			1
Nitric oxide synthase, brain					X			1
Nitric oxide synthase, inducible	X	X	X	X	X	X	X	7
Phenylethanolamine N-methyltransferase					X			1
Plasminogen activator inhibitor 1					X			1
Polyphenol oxidase 2	X	X			X		X	4
Prostaglandin F2-alpha receptor					X	X		2
Sphingosine 1-phosphate receptor 2	X	X	X	X	X		X	6
Sphingosine 1-phosphate receptor 5					X			1
Carbonic anhydrase 7	X	X						2
Corticosteroid 11-beta-dehydrogenase isozyme 1	X			X			X	3
Carbonic anhydrase 4	X	X					X	3
Steroid 17-alpha-hydroxylase/17,20 lyase	X	X	X	X			X	5
Dipeptidyl peptidase 4	X							1
Acetylcholinesterase	X	X	X	X		X	X	6
Carbonic anhydrase 6	X	X					X	3
Glucocorticoid receptor	X	X					X	3
Arachidonate 15-lipoxygenase	X	X	X				X	4

Table S3 - Broader cutoff was applied to identify targets shared by 1, 2, 3, 4, 5, 6, or all 7 compounds.

**Carbonic anhydrase 5B, mitochondrial**	X	X					X	**3**
**Corticosteroid 11-beta-dehydrogenase isozyme 2**	X	X	X	X			X	**5**
**Carbonic anhydrase 5A, mitochondrial**	X	X					X	**3**
**Aromatase**	X						X	**2**
**Androgen receptor**	X	X	X	X			X	**5**
**Dipeptidyl peptidase 8**	X							**1**
**Carbonic anhydrase 14**	X						X	**2**
**Alkaline phosphatase, tissue-nonspecific isozyme**	X	X						**2**
**Cannabinoid receptor 1**	X							**1**
**Alpha-2C adrenergic receptor**	X		X	X				**3**
**Cytochrome P450 2C19**	X	X					X	**3**
**Neuronal acetylcholine receptor subunit alpha-4**	X	X				X	X	**4**
**Carbonic anhydrase 12**	X	X					X	**3**
**Dipeptidyl peptidase 2**	X							**1**
**3-oxo-5-alpha-steroid 4-dehydrogenase 2**	X			X			X	**3**
**Toll-like receptor 9**	X							**1**
**Liver carboxylesterase 1**						X	X	**2**
**3-oxo-5-alpha-steroid 4-dehydrogenase 1**							X	**1**
**Steryl-sulfatase**							X	**1**
**Retinoic acid receptor alpha**							X	**1**
**Cocaine esterase**			X	X		X	X	**4**
**Potassium voltage-gated channel subfamily A member 3**			X				X	**2**
**Tyrosine-protein phosphatase non-receptor type 2**							X	**1**
**Carbonic anhydrase 9**		X					X	**2**
**G-protein coupled bile acid receptor 1**		X					X	**2**
**G-protein coupled receptor 35**							X	**1**
**Muscarinic acetylcholine receptor M5**							X	**1**
**Retinoic acid receptor gamma**							X	**1**
**Tyrosine-protein phosphatase non-receptor type 1**							X	**1**
**Retinoic acid receptor beta**							X	**1**
**Tubulin beta-2B chain**							X	**1**
**DNA (cytosine-5)-methyltransferase 1**							X	**1**
**NAD-dependent protein deacetylase sirtuin-2**							X	**1**

Table S3 - Broader cutoff was applied to identify targets shared by 1, 2, 3, 4, 5, 6, or all 7 compounds.

**Alpha-2B adrenergic receptor**			X					1
**Kappa-type opioid receptor**				X				1
**Squalene synthase**				X				1
**Cytochrome P450 2D6**				X				1
**Receptor-interacting serine/threonine-protein kinase 2**		X						1
**Estrogen receptor**		X						1
**Perilipin-1**		X						1
**Estrogen receptor beta**		X						1
**Prostaglandin G/H synthase 1**		X						1
**Carbonic anhydrase 13**		X						1
**Cathepsin G**			X					1
