# Supplementary material for: Computational Profiling of Monoterpenoid Phytochemicals: Insights for Medicinal Chemistry and Drug Design Strategies
Source: Int J Mol Sci. 2025 Aug 8;26(16):7671. doi: 10.3390/ijms26167671 (PMC12386793; doi:10.3390/ijms26167671)
Supplement: Supplementary file 1 [file ijms-26-07671-s001.zip › Table S4.pdf]

**Table S4** - Prediction Tools and Parameter Classification Used for ADMET and Drug-Likeness Profiling of Monoterpenoids

| ADMET Category | Parameter | References | Prediction Tools |
|---|---|---|---|
| **Physicochemical** | Molecular Weight | 100–600 Da | SwissADME, ADMETlab 2.0, Molinspiration |
| | Molecular Volume | MW/Vol | SwissADME, ADMETlab 2.0 |
| | Density | – | SwissADME |
| | Rotatable Bonds (Flexibility) | Rot/RTB | Molinspiration, SwissADME |
| | H-Bond Acceptors (nHA) | 0–12 | SwissADME, Molinspiration, ADMETlab 2.0 |
| | H-Bond Donors (nHD) | 0–7 | SwissADME, Molinspiration |
| | Topological Polar Surface Area (TPSA) | 0–140 Å² | SwissADME, Molinspiration, ADMETlab 2.0 |
| | LogP (octanol/water partition) | 0–3 | SwissADME, Molinspiration, ADMETlab 2.0 |
| | LogD (at pH 7.4) | 0–3 | ADMETlab 2.0 |
| **Absorption** | Human Intestinal Absorption (%) | >90% | pKCSM, ADMETlab 2.0, VNN-ADMET |
| | Caco-2 Permeability | >30.90 | pKCSM, ADMETlab 2.0, SwissADME |
| | Water Solubility (log S) | – | SwissADME, ADMETlab 2.0, AI Drug Lab |
| | Skin Permeability (log Kp) | -2.5 to -4.0 | ADMETlab 2.0, SwissADME |
| | P-glycoprotein Substrate/Inhibitor | No | pKCSM, ADMETlab 2.0, SwissADME |
| **Distribution** | Plasma Protein Binding (%) | <90% | ADMETlab 2.0, pKCSM, ProTox-III |
| | Volume of Distribution (VDss) (log VDss) | low < –0.15 | pKCSM, ADMETlab 2.0, SwissADME |
| | Blood-Brain Barrier (log BB) | < –1 | pKCSM, ADMETlab 2.0, SwissADME |
| | CNS Permeability (log PS) | < 3 | pKCSM, ADMETlab 2.0, VNN-ADMET |
| **Metabolism** | CYP1A2, 2C19, 2C9, 2D6, 3A4 Inhibition/Substrate | No | ADMETlab 2.0, SwissADME, AI Drug Lab, XenoSite |
| **Excretion** | Total Clearance (mL/min/kg) | high > 1.17 / low < 0.66 | pKCSM, ADMETlab 2.0 |
| | OCT & OAT Transporter Substrate/Inhibitor | No | ADMETlab 2.0, AI Drug Lab |
| **Toxicity** | Ames Mutagenicity | 0–0.3 (excellent) | ProTox-III, ToxinPRED, ADMETlab 2.0, pKCSM |
| | Hepatotoxicity | 0–0.3 (excellent) | ProTox-III, ADMETlab 2.0, VNN-ADMET |
| | Skin Sensitization | No | ToxinPRED, VNN-ADMET |
| | Carcinogenicity | No | ProTox-III, ToxinPRED, ADMETlab 2.0 |
| | hERG I & II Inhibition | No / 0.1 | PRED-hERGG, ADMETlab 2.0, ProTox-III |
| | LD50 (oral toxicity, log mg/kg) | > 0.5 / < 0.4 | ProTox-III, ADMETlab 2.0, ToxinPRED |
| | ORC (oral rat chronic) | Acceptable | ProTox-III, VNN-ADMET |
| **Medicinal Chemistry** | Quantitative Estimate of Drug-likeness (QED) | > 0.5 good / < 0.3 poor | SwissADME, ADMETopt, Molinspiration |
| | Synthetic Accessibility | > 0.42 (good) / < 0.42 (poor) | SwissADME, AI Drug Lab, ADMETopt |
| | Fraction of $sp^3$ Carbon ($Fsp^3$) | < or > 0.42 | Molinspiration, SwissADME, ADMETlab 2.0 |
| | Natural Product-likeness | >5 = good | SwissADME |
| | Lipinski's Rule Violations | 0 (Accepted) | SwissADME, ADMETlab 2.0 |
| | PAINS, Brenk, Chelator Alerts | 0 (Accepted) | SwissADME, ADMETlab 2.0 |
| | Pfizer, GSK, Golden Triangle Filters | Accepted | SwissADME, ADMETopt |
